# First-in-Human, Double-Blind, Randomized, Placebo-Controlled Trial of TQ-F3083, a New Dipeptidyl Peptidase-4 Inhibitor, in Healthy Chinese Adults

**DOI:** 10.3389/fphar.2021.689523

**Published:** 2021-07-22

**Authors:** Jingrui Liu, Xiaoxue Zhu, Hong Zhang, Haijing Wei, Deming Yang, Zhongnan Xu, Dandan Huo, Xiaojiao Li, Yanhua Ding

**Affiliations:** ^1^ Phase I Clinical Trial Unit, The First Hospital of Jilin University, Changchun, China; ^2^ Chia Tai Tianqing Pharmaceutical Group Co., Ltd., Nanjing, China

**Keywords:** TQ-F3083, type 2 diabetes, phase I, pharmacokinetics, pharmacodynamics

## Abstract

**Background:** As a novel dipeptidyl peptidase-4 (DPP-4) inhibitor, TQ-F3083 represents a promising new drug for type 2 diabetes mellitus (T2DM). This phase I, first-in-human study evaluated the tolerability, pharmacokinetics, and pharmacodynamics of TQ-F3083 in healthy Chinese adults.

**Methods:** Sixty healthy participants total were enrolled in the single-ascending dose, multiple-dose, and food-effect studies. Safety endpoints included adverse events (AEs), vital signs, 12-lead electrocardiogram, abdominal ultrasound, chest X-ray, physical examination, and clinical laboratory tests. Blood, urine, and feces samples were collected for pharmacokinetic analyses. Pharmacodynamic parameters were evaluated based on DPP-4 activity and the active glucagon-like peptide-1 concentration.

**Results:** In total, 22 treatment-related AEs, mostly grade 1 or 2, were reported in 14 individuals. No deaths, serious AEs, or grade ≥4 AEs occurred, and no dose-dependent AEs were demonstrated. For pharmacokinetic characteristics, dose linearity was analyzed using power model. The slopes (90% CIs) were 1.08 (1.02–1.13) and 1.05 (0.99–1.11) for AUC_0-t_ and AUC_0-∞_, suggesting liner pharmacokinetic characteristic after oral dose TQ-F3083 from 2 to 160 mg. The accumulation factor was 1.39 after multiple dose for 7 days. Decreased plasma exposure (84.87% decrease in C_max_, 49.23% in AUC_0-t_, and 47.77% in AUC_0-∞_) was observed with administration after a high-fat and high-calorie standardized breakfast. The 0–72 h TQ-F3083 excretion recovery percentages were 7.84% in urine and 5.76% in feces. Over 80% DPP-4 inhibition for 24 h was observed in the 20–160 mg cohorts, and the model-estimated 50% effective concentration was 1.10 ng/ml. The concentration of active glucagon-like peptide-1 increased after TQ-F3083 administration, but no obvious dose dependency was observed.

**Conclusion:** TQ-F3083 was well tolerated in healthy Chinese adults, and the pharmacokinetic and pharmacodynamic characteristics support further evaluation of TQ-F3083 in a trial in T2DM patients.

## Introduction

Diabetes mellitus (DM) is a complex, progressive global epidemic with an estimated current prevalence of 6.4%, and over 693 million people are estimated to have DM by 2045 (Guariguata et al., 2014; Cho et al., 2018). Type 2 DM (T2DM) accounts for 90–95% of all cases, and the incidence continues to increase worldwide with the aging of the population and adoption of unhealthy diets (Li et al., 2018). Microvascular and macrovascular complications of T2DM occur frequently and are associated with a significant reduction in life expectancy, and research has determined that achieving and maintaining glycemic control is the key treatment goal for preventing complications of T2DM (Holman et al., 2008; Ahrén et al., 2017). Despite the existence of a wide range of treatment options, a high proportion of T2DM patients still fail to achieve the recommended target glycated hemoglobin (HbA1c) level of less than 7.0% (Stark Casagrande et al., 2013; Rosenstock et al., 2019). Currently, metformin is the first-line treatment agent prescribed for most T2DM patients based on its favorable efficacy, safety, and low cost; however, when initial metformin monotherapy fails, another antihyperglycemic drug that acts *via* different mechanism is added (Nathan et al., 2009; Davies et al., 2018). Conventional second-line therapies including sulfonylureas, thiazolidinediones, alpha glucosidase inhibitors, and insulin have limited long-term efficacy and can cause various side effects such as hypoglycemia, swelling, bloating, and flatulence (Kerru et al., 2018). Thus, the need for novel antihyperglycemic drugs with better efficacy and safety profiles persists.

Dipeptidyl peptidase-4 (DPP-4) inhibitors are incretin-based antihyperglycemic drugs that block DPP-4–mediated degradation of the incretin hormones glucagon-like peptide-1 (GLP-1) and glucose-dependent insulinotropic polypeptide. Clinical studies have confirmed that DPP-4 inhibitors provide distinct improvement of glycemic control and present a favorable safety profile without body weight gain or an increased risk of hypoglycemia (Brazg et al., 2007; Arechavaleta et al., 2011; Nauck, 2016; Frias et al., 2019). Several DPP-4 inhibitors have been approved for clinical use or are still in development including peptidomimetic and non-peptidomimetic inhibitors. Unfortunately, many of these agents are excreted through the kidneys and, thus, are not suitable for patients with advanced T2DM (Moon et al., 2020). Linagliptin exhibits distinct pharmacological characteristics from other DPP-4 inhibitors, in that it is the only agent mainly excreted as parent drug through the intestinal tract, and thus, no dose adjustment is required in patients with hepatic or renal insufficiency. But linagliptin shows a long terminal half-life of 142 h, accumulation may be a challenge with long-term oral administration (Hüttner et al., 2008; Heise et al., 2009; Blech al., 2010; Sarashina et al., 2010).

TQ-F3083 is a highly selective DPP-4 inhibitor that is currently in development for the treatment of T2DM. It has been patented with the patent number CN201110154294.X. The pharmacology of TQ-F3083 has been extensively investigated in preclinical models and the results obtained (unpublished data) have been submitted to the Center for Drug Evaluation, China Food, and Drug Administration (CFDA) with the application for clinical study approval in humans that was ultimately granted. Briefly, preclinical pharmacokinetics (PK) was evaluated in Wistar rats and Cynomolgus monkeys, and the results showed no obvious accumulation after multiple oral administration TQ-F3083 for 7 days in both species. Metabolite identification was conducted using UPLC/Q-TOF MS method in human liver microsomes and the effect of specific CYP450 enzymes inhibitors on TQ-F3083 metabolism were explored. The results showed metabolism of TQ-F3083 was mainly mediated by CYP450 enzymes, and CYP3A was the main metabolic enzyme. Tissue distribution, metabolism, and excretion research was conducted in Wistar rats. After oral administration of 9 mg/kg, TQ-F3083 was widely distributed in the tissues and the concentration in most tissues was higher than that in plasma. The main distribution organs were kidney, liver, lung, pancreas, bladder, and digestive tract. The concentration in brain was much lower than that in other tissues and plasma, indicating that it has a low degree of penetration through the blood-brain barrier. The parent drug was the main substance found in plasma, urine, feces, and bile samples. In addition, acetylated metabolite M6 was detected in plasma and the peak area of M6 was less than 10% that of parent drug. Metabolites including M6, single oxidation and dehydrogenation metabolite M2-3, single oxidation metabolite M3, double oxidation and hydrogenation metabolite M4, double oxidation and dehydrogenation metabolite M5 were also identified in urine, feces, and bile. In excretion study, TQ-F3083 and metabolite M6 were detected. The 0–96 h excretion rates (TQ-F3083 and M6) were 26.4% in feces and 13.3% in urine, and the parent drug accounted for 23.8 and 12.8% respectively in feces and urine. CYP450 enzymes inhibition and induction study showed that TQ-F3083 had no induction to CYP450 enzymes and weak inhibition were observed on CYP2C9 and CYP3A4. Ultracentrifugation method was used to determine the human plasma protein binding rate of TQ-F3083. At the TQ-F3083 concentrations of 50, 200, and 800 ng/ml, the plasma protein binding rate were 76.3, 72.7 and 71.3%, respectively. Preclinical pharmacodynamic (PD) evaluations conducted in an obese mice model of T2DM determined the minimum effective dose to be 3 mg/kg, which translated into a human equivalent dose of 0.25 mg/kg according to body surface area. Toxicology studies indicated that the No Observed Adverse Effect Levels (NOAELs) are 292 mg/kg (AUC_0-t_ at NOAEL: 52,000 h*ng/mL) for Sprague–Dawley rats and 69 mg/kg (AUC_0-t_ at NOAEL: 44,900 h*ng/mL) for Cynomolgus monkeys. Here we present the first in-human study investigating the tolerability, PK, and PD of TQ-F3083 in healthy Chinese adults. A dose range of 2–160 mg was selected for use in the clinical trial based on preclinical studies of the drug’s activity and toxicity.

## Materials and Methods

### Subjects

Healthy Chinese adults were screened and enrolled in the study conducted in the First Hospital of Jilin University, China. The eligibility criteria were: general healthy condition; male or female; age 18–65 years; minimum body weights of 50 and 45 kg for males and females, respectively; body mass index 18–28 kg/m^2^; and no history of cardiac, hepatic, renal, gastrointestinal, or neurologic diseases. All participants were required to practice birth control and have no plan to conceive during the subsequent 6 months. The exclusion criteria were: smoking more than five cigarettes a day during the 3 months before the study; enrollment in other trials in the preceding 3 months; any clinically significant abnormality on a laboratory test or abnormal 12-lead electrocardiogram (ECG), chest X-ray, or abdominal ultrasound in screening; a history of drug abuse and/or alcoholism; intake of any other drugs, vitamins, or herbal medicine within 14 days or intake of any drugs known to influence the activity of drug-metabolizing enzymes within 28 days; pregnant or breastfeeding; multiple food and drug allergies; and intolerability of high-fat and high-calorie standardized breakfast (only for food-effect cohorts). The study was approved by CFDA (approval number: 2016L10560) and the ethics committee of the First Hospital of Jilin University. It was conducted under the guidelines of the Declaration of Helsinki and the Principles of Good Clinical Practice. Written informed consent was obtained from all participants or their legal representatives prior to their enrollment.

### Study Design

**FIGURE 1:**
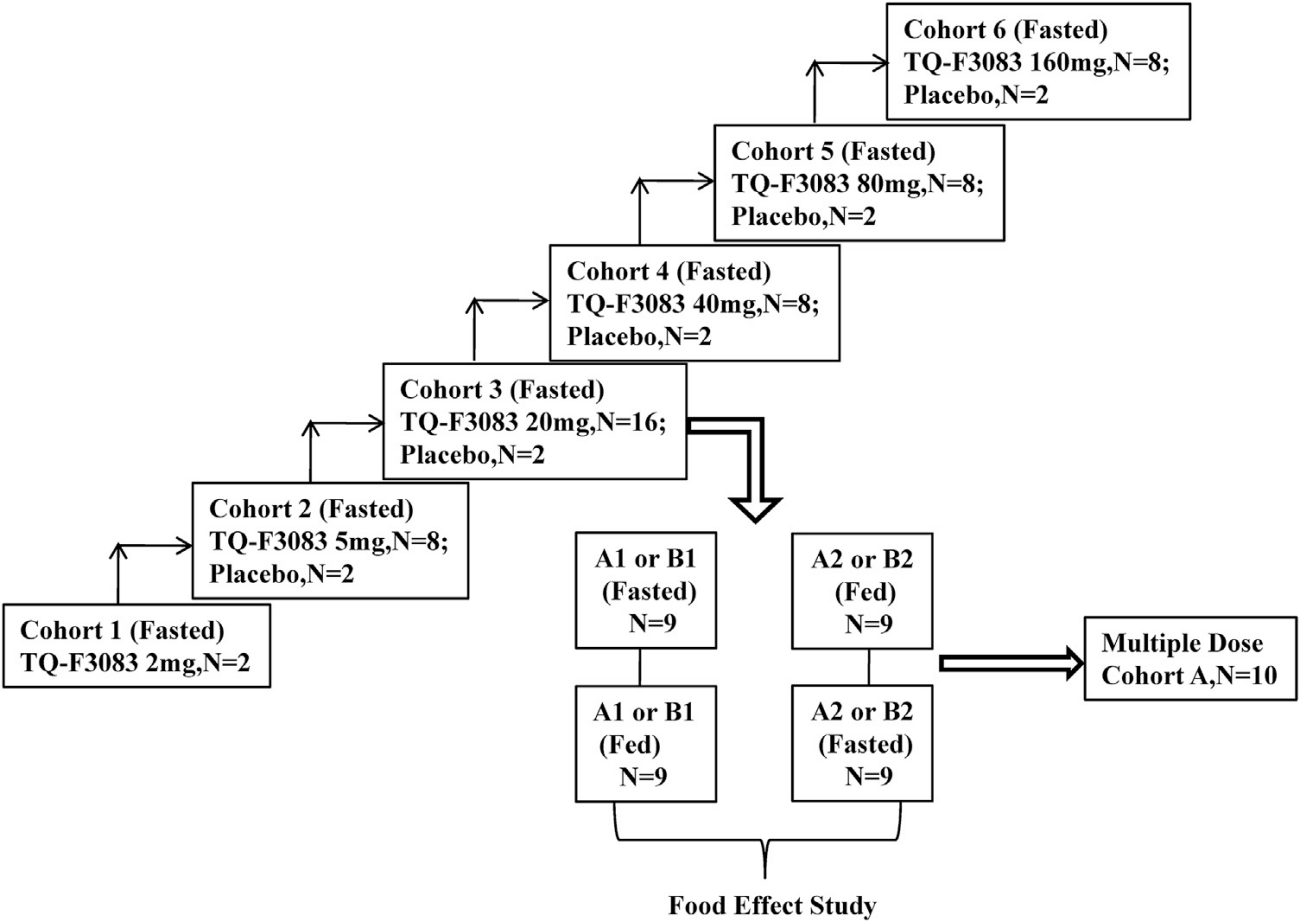
Study flowchart.

This phase I, double-blind, randomized, placebo-controlled, dose-escalation study was conducted at First Hospital of Jilin University, China (registration no: ChiCTR-IIR-17013561 registered at http://www.chictr.org.cn/). TQ-F3083 capsules and the corresponding placebo were produced and supplied by Chia Tai Tianqing Pharmaceutical Group Co., Ltd. in unit doses of 2 and 5 mg. The compound would be made available to bona fide scientists looking to verify this work. Six cohorts were designed in the study for testing of the following dosages: 2, 5, 20, 40, 80, and 160 mg (cohorts 1–6). Two subjects were enrolled into cohort 1 and given an single TQ-F3083 capsule orally after an overnight fast. Cohorts 2 and 4–6 had 10 participants in each and were used to conduct a single-dose study. These participants were randomly given TQ-F3083 or placebo at a ratio of 4:1 after an overnight fast. Cohort 3 included 18 participants, and they were randomized to groups A and B. Single-dose, multiple-dose, and food-effect studies were conducted with group A, and food-effect and metabolic transformation studies were conducted with group B. Group A consisted of 10 participants divided equally into two groups to receive treatment in two sequences for a two-period, crossover food-effect study: A1 (fasted-fed) and A2 (fed-fasted), and randomized to TQ-F3083 or placebo at a ratio of 4:1. The washout period was 7 days for every single dose, and after that, multiple doses were administered once a day for 7 days after overnight fasting from days 15–21. The study in group A in the fasted state was regarded as single-dose study of the TQ-F3083 20 mg cohort. All eight participants in group B were given TQ-F3083 in two sequences, B1 (fasted-fed) and B2 (fed-fasted), with a 7-days washout period. Urine and feces samples were collected from participants in group B for metabolic transformation analysis after administration of a single dose in the fasted state. Whether dose escalation to the next dose level was decided only after reviewing the safety and tolerability results of all subjects in the previous cohort (Figure 1). The proportions of male and female participants needed to be close in each cohort, and administration of each successive dose depended on the safety and tolerability of previous doses.

Dose-limiting toxicities (DLTs) were evaluated according to the National Cancer Institute (NCI) Common Terminology Criteria for Adverse Events (CTCAE) version 4.03 and defined as the emergence of the following treatment-related adverse events (AEs): more than half of participants suffered Grade 3–4 treatment-related toxicities or one subject experienced a treatment-related serious adverse event (SAE).

The primary objectives of the trial were to evaluate the safety and tolerability of TQ-F3083 as well as to determine the DLTs and maximum tolerated dose (MTD) of TQ-F3083. The secondary objectives included characterization of the PK, PD, food-effect, and metabolic transformation characteristics of TQ-F3083.

### Safety Analysis

Safety was assessed according to the NCI CTCAE version 4.03 including adverse events (AEs), vital signs, 12-lead ECG, abdominal ultrasound, chest X-ray, physical examination, and clinical laboratory tests. Abdominal ultrasound and chest X-ray were only performed during screening. For cohorts 1, 2, and 4–6, physical examination and clinical laboratory tests were conducted during screening and on day 4; vital signs were measured during screening, at admission, pre-dose, and at 2, 4, 6, 10, 24, 72, and 120 (except for cohort 1) h after dosing; 12-lead ECGs were measured during screening, pre-dose and 2, 4, 6, 10, 24, and 72 h after dosing. For group A in cohort 3, physical examination was conducted during screening and at days 4, 11, and 24; clinical laboratory tests were conducted during screening and at days 4, 11, 18, 21, and 24; vital signs were measured during screening, admission, pre-dose and 2, 4, 6, 10, 24, 72, and 120 h after dosing on days 1, 8, and 21, pre-dose and 2, 4, 6, 10, 24, and 48 h on day 18, and pre-dose on days 15–17; 12-lead ECGs were measured during screening, pre-dose and 2, 4, 6, 10, 24, and 72 h on days 1, 8, 18, and 21. For group B, physical examination and clinical laboratory tests were conducted during screening and on days 4 and 11; vital signs were measured during screening, on admission, pre-dose and 2, 4, 6, 10, 24, 72, and 120 h after dosing on days 1 and 8; and 12-lead ECGs were measured during screening, pre-dose and at 2, 4, 6, 10, 24, and 72 h on days 1 and 8.

### PK Sampling and Analysis

Blood samples (4 ml) for PK analyses were collected in tubes containing K_2_EDTA anticoagulant. The sampling time points were pre-dose and 0.25, 0.5, 0.75 (except for cohort 1), 1, 1.5, 2, 3, 4, 6, 8, 12, 24, 48, 72, and 120 (except for cohort 1) h post-dose for cohorts 1, 2, and 4–6. For cohort 3, the same sampling time points were performed on days 1, 8, and 21 (only for group A) and pre-dose on days 19 and 20 (only for group A). Blood samples were centrifuged at 3,000 rpm for 10 min at 2–8°C and stored at –80°C until analysis by high performance liquid chromatography/mass spectrometry (HPLC-MS/MS).

Urine and feces samples were collected after fasting in group B with the time intervals of sampling 0, 0–6, 6–12, 12–24, 24–48, 48–72, and 0–72 h. Samples were stored at –80°C until analysis.

Plasma PK data were analyzed by standard non-compartmental methods using WinNonlin version 7.0 (Certara, Princeton, NJ, United States), and the following PK parameters were calculated: peak plasma concentration (C_max_), time to C_max_ (T_max_), area under the curve (AUC) from time 0 to the last measurable concentration timepoint (AUC_last_), AUC from time 0 to infinity (AUC_0-∞_), terminal elimination half-life (t_1/2_), clearance (CL/F), apparent volume of distribution (V_z_/F), and each of these parameters at steady-state: C_max,ss_, C_min,ss_, T_max,ss_, AUC_last,ss_, AUC_0- ∞,ss_, CL/F_ss_, V_z_/F_ss_, and accumulation ratio (R_ac_), based on AUC_0–24_ of day 21 vs. the first fasted dose. For urine and feces data, the accumulative excretion (Ae_u0-72_ and Ae_f0-72_) and the ratios (Ae_u0-72_% and Ae_f0-72_%) were calculated.

### Bioanalysis

Bioanalysis was performed at Covance Inc. (Shanghai, China). High-performance LC (Waters, Acquity, UPLC)-MS/MS (Sciex API 5500) was used to quantify the concentrations of TQ-F3083 in plasma, urine and feces samples. The chromatography system consisted of a Waters, Xbridge C18, 50 mm × 2.1 mm, 3.5 mm particle size analytic column. The analytes were eluted using a 0.1% (vol/vol) formic acid with 5 mM ammonium formate in water and 0.1% (vol/vol) formic acid in acetonitrile:methanol (vol:vol 1:1). Protein precipitation was employed for plasma processing, and liquid–liquid extraction for urine and feces samples. The mass spectrometer was operated in the positive electrospray ionization mode and multiple reaction monitoring was used to detect transitions at m/z (M + H)+ 466.2/246.2 for TQ-F3083. TQ-F3083 concentrations were determined using appropriate calibration curves obtained from standards in the range of 0.1–100 ng/ml. The precision (coefficient of variation) and accuracy (relative error) of the analysis method were 0.6–18.2% and −4.9%–5.0%, respectively in plasma, 1.3–3.7% and −1.2%–1.8% in urine, 3.7–7.2% ,and −3.3%–8.0% in feces, which are acceptable for the analytical standard of ≤15% (20% at the lower limit of quantification). A total of 136 samples were re-analyzed after being diluted because of the higher concentration than the upper limit of quantification 100 ng/ml.

### PD Analysis

The PD characteristics of TQ-F3083 were assessed based on the concentrations of insulin, C-peptide, glucagon and active GLP-1 in plasma, as well as on DPP-4 activity. The concentrations of insulin and C-peptide were measured using a validated Enzyme-linked immunosorbent assay (ELISA) method with commercial insulin assay kit (ALPCO, United States) and C-peptide assay kit (ALPCO, United States). Microplate reader (Molecular Devices, SpectraMax M5e, CA, United States) was used in the methods with the quantitation range 15.6–2000 pmol/L for insulin and 30–2,400 pmol/L for C-peptide. The concentrations of glucagon and active GLP-1 were measured using a validated electrochemiluminescence (ECL) method with commercial glucagon assay kit (MSD, United States) and active GLP-1 assay kit (MSD, United States). ECL analyzer (MESO QUICKPLEX TM SQ 120) was used in the methods with the quantitation range 45.0–951 pg/ml for glucagon and 0.58–200 pg/ml for active GLP-1. DPP-4 activity was measured using a validated fluorometric method with a commercial DPP-4 activity assay kit (BACHEM, Switzerland). DPP-4 cleaves the substrate H-Gly-Pro-AMC to release a quenched fluorescent group AMC, which was detected at an excitation wavelength of 360 nm and emission wavelength of 465 nm (Molecular Devices, SpectraMax M5e, CA, United States) with the quantitation range 31.3–1,500 ng/ml. Blood samples (2 and 4 ml) were collected for measurement of glucagon, active GLP-1, insulin, and C-peptide with the sampling time points of pre-dose, 0.5, 1, 2, 4, 6, 12, and 24 h post-dose for cohorts 1, 2, and 4–6. For group A in cohort 3, these time points were pre-dose, 0.5, 1, 2, 4, 6, 12, and 24 h post-dose on days 1, 8, and 21 followed by pre-dose on days 15, 17, and 19. Two-milliliter blood samples were needed to test the activity of DPP-4 at the sampling time points of pre-dose, 0.5, 1, 6, 12, 24, 48, and 72 h post-dose for cohorts 1, 2 and 4–6. For group A in cohort 3, samples were collected at pre-dose, 0.5, 1, 6, 12, 24, 48, and 72 h post-dose on days 1, 8, and 21, followed by pre-dose on days 15, 17, and 19. Descriptive statistics, a GLP-1 concentration–time curve and a DPP-4 inhibition ratio (the percentage of inhibited DPP-4)–time curve were employed to analyze the PD characteristics of TQ-F3083.

### PK/PD Analysis

To evaluate the relationship between the TQ-F3083 plasma concentration and DPP-4 inhibition ratio, data were modeled using the E_max_ model with the Hill slope (γ) fixed to 1: DPP4 inhibition ratio = E_max_*C^γ^/(EC_50_^γ^+C^γ^), where E_max_ is the maximal inhibition ratio; EC_50_ is the 50% TQ-F3083 effective plasma concentration; and C is the plasma concentration of TQ-F3083.

### Concomitant Medications

No concomitant medications were allowed during the study except for the treatments of AEs. When mild AEs emerged, no medications were recommended, whereas for moderate or more severer AEs, symptomatic treatments were administered. Any use of potent inhibitors and inducers of hepatic metabolic enzymes was prohibited, and close monitoring was performed for any patients taking a CYP2C9 substrate.

### Statistical Analysis

Statistical analyses were performed using SAS software, version 9.4 (SAS Institute Inc., United States). Descriptive statistics were used to summarize continuous variables as cases, means with standard deviations, medians, quartiles, maximum, and minimum. For categorical variables, frequencies and percentages were calculated. A regression power model, relating log-transformed C_max_ and AUC parameters to log-transformed dose, was used to investigate dose proportionality. Food-effect PK parameters were analyzed using a mixed effects model with period, sequence, and treatment as fixed effects and subjects as the random effect. Logarithmic transformations of C_max_, AUC_0-∞_, and AUC_last_ were performed to calculate the 90% confidence intervals (CIs) of the geometric mean ratio (GMR) between fasting and fed administration. If the calculated 90% CI values fell entirely within the range of 0.80–1.25, no significant food-effect was considered.

## Results

### Demographics and Baseline Data

After screening of a total of 281 adults, 60 participants were enrolled in the present dose-escalation study. All participants completed the entire study and were included in the safety analyses. Fifty participants who received TQ-F3083 were included in the PK analyses and sixty participants were included in the PD analyses. The demographic characteristics and baseline clinical data of the study participants are presented in Table 1.

**TABLE 1:** Summary of study participants’ demographic and basic clinical characteristic.

Characteristic	2 mg	5 mg	20 mg (group A)	20 mg (group B)	40 mg	80 mg	160 mg	Placebo
	(*n* = 2)	(*n* = 8)	(*n* = 8)	(*n* = 8)	(*n* = 8)	(*n* = 8)	(*n* = 8)	(*n* = 10)
Age, mean (SD), years	27.00 (0)	37.25 (6.59)	34.00 (3.57)	34.00 (5.94)	34.38 (8.87)	32.88 (7.69)	36.50 (5.22)	32.10 (7.99)
Sex, n (%)								
Male, n (%)	2 (100.00)	3 (37.50)	4 (50.00)	4 (50.00)	3 (37.50)	3 (37.50)	3 (37.50)	9 (90.00)
Female, n (%)	0	5 (62.50)	4 (50.00)	4 (50.00)	5 (62.50)	5 (62.50)	5 (62.50)	1 (10.00)
Height, mean (SD), cm	164.60 (4.10)	160.46 (7.86)	161.68 (8.33)	162.38 (7.23)	160.26 (9.05)	161.98 (8.59)	164.49 (7.00)	167.52 (6.73)
Weight, mean (SD), kg	66.35 (6.25)	60.76 (5.65)	62.10 (9.01)	57.34 (9.96)	61.76 (5.99)	58.98 (6.87)	66.14 (6.49)	63.20 (7.34)
BMI, mean (SD), kg/m^2^	24.42 (1.09)	23.66 (2.11)	23.80 (3.22)	21.64 (2.62)	24.07 (1.77)	22.49 (2.27)	24.44 (1.77)	22.59 (2.84)

SD, standard deviation; BMI, body mass index.

### Safety and Tolerability

Overall, TQ-F3083 was well-tolerated in all study cohorts with no DLTs reported. The favorable safety results prevented MTD identification in the present study. The relevant data for all treatment-related AEs are presented in Table 2. In total, 22 treatment-related AEs were reported in 14 (23.33%) participants, and most treatment-related AEs were scored as grade 1 or 2 and resolved without treatment. Two participants experienced grade 3 elevation of triglyceride level. Both were included in cohort 2, with one receiving oral administration of TQ-F3083 and the other receiving placebo, and both recovered without treatment. No deaths, SAEs, or grade ≥4 AEs were reported in any cohort.

**TABLE 2:** Summary of treatment-related AEs (NCI CTCAE grades) among cohorts given TQ-F3083 at doses of 2–160 mg.

Cohorts	2 mg (*n* = 2)		5 mg (*n* = 8)		20 mg Fasted (*n* = 16)		20 mg Fed (*n* = 16)		20 mg MD (*n* = 8)		40 mg (*n* = 8)		80 mg (*n* = 8)		160 mg (*n* = 8)		Placebo (*n* = 10)	
Item, n (%)	Grade 1–2	Grade3	Grade 1–2	Grade 3	Grade 1–2	Grade3	Grade 1–2	Grade3	Grade 1–2	Grade3	Grade 1–2	Grade3	Grade 1–2	Grade3	Grade 1–2	Grade3	Grade 1–2	Grade 3
Dizziness	0	0	0	0	4 (25.0)	0	0	0	0	0	0	0	0	0	0	0	0	0
Fatigue	0	0	0	0	4 (25.0)	0	0	0	0	0	0	0	0	0	0	0	0	0
Elevated thyrotropin	1 (50.0)	0	0	0	1 (6.25)	0	0	0	1 (12.5)	0	0	0	0	0	0	0	0	0
Elevated triglyceride	0	0	0	1 (12.5)	1 (6.25)	0	0	0	0	0	0	0	0	0	0	0	0	1 (10.0)
Lymphocytopenia	0	0	0	0	0	0	1 (6.25)	0	0	0	0	0	0	0	0	0	0	0
Urine erythrocyte positive	0	0	0	0	1 (6.25)	0	0	0	0	0	0	0	0	0	0	0	0	0
Neutrophilic granulocytosis	0	0	0	0	0	0	0	0	0	0	1 (12.5)	0	0	0	0	0	0	0
Leukocytosis	0	0	0	0	0	0	0	0	0	0	1 (12.5)	0	0	0	0	0	0	0
Urinary tract infection	0	0	0	0	0	0	0	0	0	0	0	0	0	0	1 (12.5)	0	0	1 (10.0)
Ventricular extrasystole	0	0	0	0	0	0	0	0	0	0	0	0	0	0	1 (12.5)	0	0	0
Supraventricular arrhythmia	0	0	0	0	0	0	0	0	0	0	0	0	0	0	1 (12.5)	0	0	0

NOTE: No treatment-related grade 4–5 AEs were observed in any of the single ascending dose cohorts.

NCI CTCAE, National Cancer Institute Common Terminology Criteria for Adverse Events; MD, Multiple dose.

Dizziness (8.0%, *n* = 4), fatigue (8.0%, *n* = 4), and elevated thyrotropin (6.0%, *n* = 3) were the most frequently reported treatment-related AEs, and the incidence of each was greater in the treatment group than in the placebo group. All of these AEs were rated as grade 1. No apparent increases in AEs was observed with the administration of multiple doses or dosing after a high-fat and high-calorie standardized breakfast, and no dose-dependent AEs were observed.

### PK Properties

**FIGURE 2:**
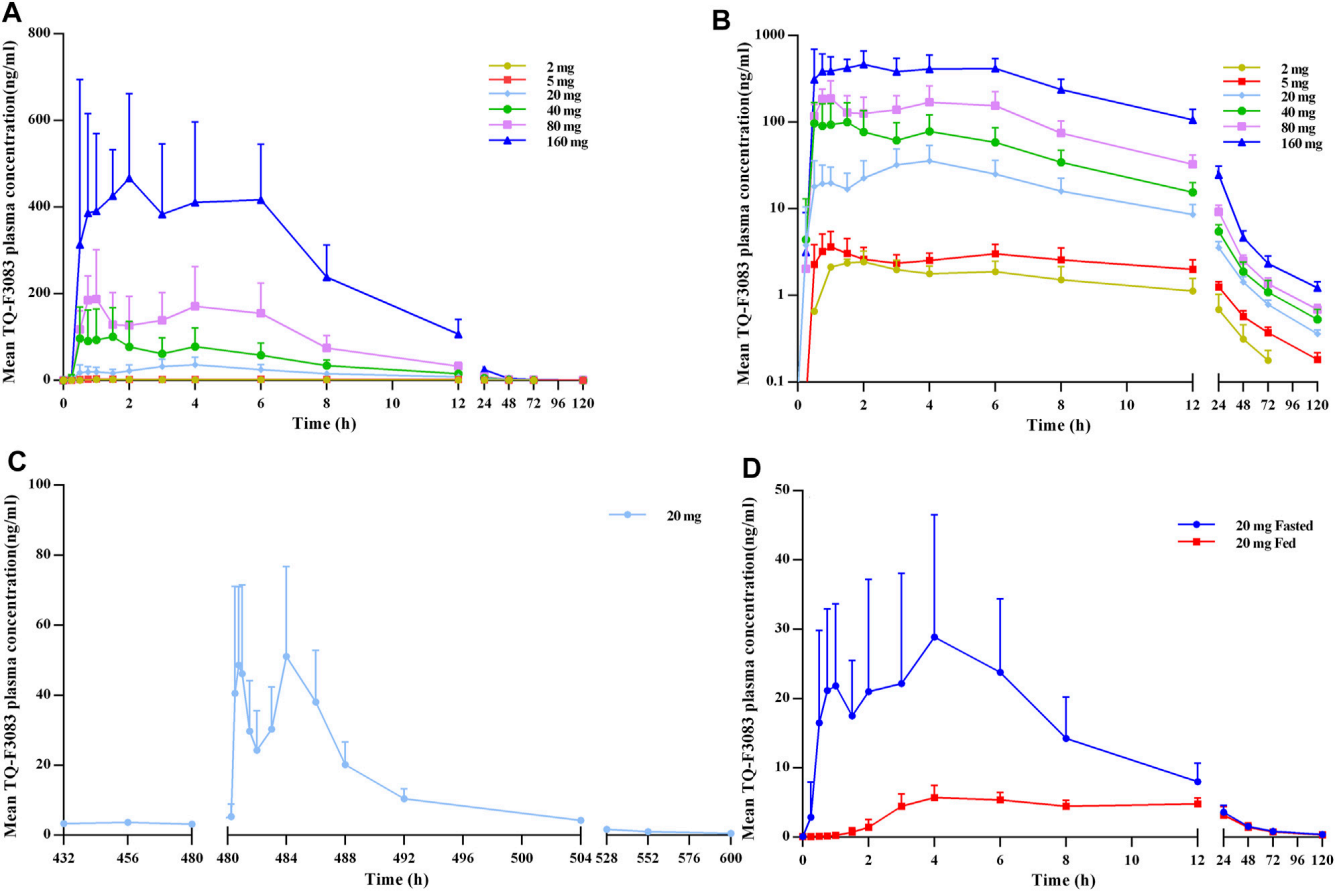
TQ-F3083 mean (SD) plasma concentration–time profiles after administration of a single ascending dose from 2 to 160 mg. **(A)**, mean values after single dose. **(B)**, profiles plotted on a semi-logarithmic scale. **(C)**, mean values after multiple doses of 20 mg. **(D)**, mean values after single dose of 20 mg in the fasted and fed states.

The plasma PK parameters after administration of a single dose of TQ-F3083 over the dosage range of 2–160 mg are presented in Table 3. The mean TQ-F3083 plasma concentration–time profiles after single ascending dose (SAD) administration are shown in Figures 2A,B. After a single dose of oral administration, TQ-F3083 was rapidly absorbed with a median T_max_ range of 0.88–3.50 h. The mean t_1/2_ values were 25.52–41.62 h, not prolonging according to increasing dosages. The AUC_0-t_, AUC_0-∞_, and C_max_ for TQ-F3083 increased dependent on dose, and the relationships between dosages and PK parameters were analyzed using power model. The slopes (90% CIs) were 1.08 (1.02–1.13) for ln AUC_0-t_ and 1.05 (0.99–1.11) for AUC_0-∞_, suggesting a clear linear characteristic with an oral dose of TQ-F3083 from 2 to 160 mg. For C_max_, the slope (90% CI) was 1.48 (1.33–1.50), showing a higher proportional increase than the dose increase. The CL/F ranged from 39.62 to 49.48L/h, and no dose-dependent changes were observed. The V_z_/F ranged from 1,510.12–2,920.35 L with no dose-dependent changes observed. The large volume of distribution indicated that TQ-F3083 was mainly distributed in tissues.

**TABLE 3:** Pharmacokinetic (PK) properties of TQ-F3083 after administration of a single ascending dose of 2, 5, 20, 40, 80, and 160 mg.

PK parameter	Dosage					
	2 mg (*n* = 2)	5 mg (*n* = 8)	20 mg (*n* = 8)	40 mg (*n* = 8)	80 mg (*n* = 8)	160 mg (*n* = 8)
T_max_, median (min–max), h	1.75 (1.50–2.00)	1.00 (0.50–6.00)	3.50 (0.50–6.00)	0.88 (0.50–4.00)	3.00 (0.75–6.00)	2.00 (0.50–6.00)
C_max_, mean (SD), ng/mL	2.61 (0.57)	4.08 (1.74)	42.11 (12.89)	136.69 (59.47)	258.38 (86.91)	678.63 (201.79)
AUC_0-24_, mean (SD), h*ng/mL	30.14 (10.63)	49.25 (13.15)	310.18 (104.74)	726.66 (299.61)	1,497.95 (488.21)	4,245.96 (638.09)
AUC_0-t_, mean (SD), h*ng/mL	47.39 (18.27)	94.06 (19.28)	418.33 (118.11)	879.13 (320.47)	1714.13 (489.62)	4,696.99 (662.19)
AUC_0-∞_, mean (SD), h*ng/mL	53.79 (18.87)	105.12 (21.61)	437.25 (118.26)	908.54 (329.96)	1754.24 (489.58)	4,761.54 (659.83)
T_1/2_, mean (SD), h	25.52 (5.11)	41.62 (6.09)	36.35 (2.24)	37.24 (10.23)	40.14 (4.97)	35.87 (10.02)
CL/F, mean (SD), L/h	39.62 (13.90)	49.03 (8.26)	48.57 (12.14)	49.48 (19.75)	49.23 (15.19)	34.22 (5.15)
V_z_/F, mean (SD), L	1,510.12 (804.16)	2,920.35 (548.09)	2,571.76 (762.20)	2,603.01 (1,069.33)	2,814.63 (767.35)	1778.19 (590.22)

T_max_, time to peak plasma concentration; C_max_, peak plasma concentration; AUC_0-24_, area under the plasma concentration-time curve from time zero to 24 h; AUC_0-t_, area under the plasma concentration-time curve from time zero to time t; AUC_0-∞_, area under the plasma concentration-time curve from time zero to infinity; T_1/2_, terminal elimination half-life; CL/F, apparent clearance; V_z_/F, apparent volume of distribution; SD, standard deviation.

The plasma PK parameters after administration of multiple doses of 20 mg TQ-F3083 for 7 days are presented in Table 4. Figure 2C shows the mean TQ-F3083 plasma concentration–time profiles after administration of multiple doses. Visual inspection of the TQ-F3083 trough concentration data indicated that steady-state was reached after approximately 4 days of continuous administration. The accumulation factor R_ac_ was 1.39 (calculated by AUC_tau,ss_/AUC_0-24_ for a single dose), showing no obvious accumulation after multiple doses had been taken. The t_1/2,ss_, CL/F_ss_ and V_z_/F_ss_ were 42.20 h, 50.42 L/h and 3061.57 L, respectively, which were similar to these parameters for single dose administration. The plasma PK parameters for TQ-F3083 administered in the fasted vs. fed state are presented in Table 5. Figure 2D shows the mean TQ-F3083 plasma concentration–time profiles obtained when the participants were in a fasted vs. fed state. Following a high-fat and high-calorie standardized breakfast, there was an 84.87% decrease in the TQ-F3083 C_max_, a 49.23% decrease in the AUC_0-t_ and a 47.77% decrease in the AUC_0-∞_. The inter-individual variation of main PK parameters was similar between the fasted and fed state. From the mixed effects model analysis, none of the calculated 90% CI values for AUC_0-t_, AUC_0-∞_ and C_max_ fell entirely within the range of 0.80–1.25 (Table 6), which indicated significantly decreased absorption when TQ-F3083 was taken after a high-fat and high-calorie standardized breakfast. The median T_max_ was prolonged from 3.50 h in the fasted state to 4.00 h in the fed state. The t_1/2_ was 34.98 h in the fed state, which was comparable to that in the fasted state. The CL/F increased from 52.09 to 97.24 L/h from the fasted to fed state and the V_z_/F increased from 2,881.49 to 4,866.60 L.

**TABLE 4:** Pharmacokinetic (PK) properties of TQ-F3083 after administration of multiple 20-mg doses.

PK parameter	Dosage
	20 mg (*n* = 8)
T_max,ss_, median (min–max), h	4.00 (0.50–6.00)
C_max,ss_, mean (SD), ng/mL	73.81 (18.06)
C_min,ss_, mean (SD), ng/mL	3.16 (0.31)
AUCtau,ss, mean (SD), h*ng/mL	414.78 (96.84)
AUC_0-t,ss_, mean (SD), h*ng/mL	549.35 (105.69)
AUC_0-∞,ss_, mean (SD), h*ng/mL	580.87 (109.85)
T_1/2,ss_, mean (SD), h	42.20 (3.55)
CL/F_ss_, mean (SD), L/h	50.42 (11.00)
V_z_/F_ss_, mean (SD), L	3061.57 (721.09)
R_ac_, mean (SD)	1.39 (0.24)

T_max,ss_, time to peak plasma concentration at steady state; C_max,ss_, peak plasma concentration at steady state; C_min,ss_, minimum plasma concentration at steady state; AUC_tau,ss_, area under the plasma concentration-time curve over a dosing interval at steady state; AUC_0-t,ss_, area under the plasma concentration-time curve from time zero to time t at steady state; AUC_0-∞,ss_, area under the plasma concentration-time curve from time zero to infinity at steady state; T_1/2,ss_, terminal elimination half-life at steady state; CL/F_ss_, apparent clearance at steady state; V_z_/F, apparent volume of distribution; R_ac_, accumulation ratio; SD, standard deviation.

**TABLE 5:** Pharmacokinetic (PK) properties of TQ-F3083 after administration of a single dose of 20 mg in the fasted and fed state.

PK Parameter	Dosage	
	20 mg fasted (*n* = 16)	20 mg fed (*n* = 16)
T_max_, median (min–max), h	3.50 (0.50–6.00)	4.00 (3.00–24.00)
C_max_, mean (SD), ng/mL	41.22 (14.32)	6.24 (1.51)
AUC_0-24_, mean (SD), h*ng/mL	277.79 (93.08)	95.54 (15.19)
AUC_0-t_, mean (SD), h*ng/mL	392.82 (116.43)	199.44 (55.81)
AUC_0-∞_, mean (SD), h*ng/mL	415.22 (120.40)	218.23 (65.71)
T_1/2_, mean (SD), h	37.85 (4.17)	34.98 (8.32)
CL/F, mean (SD), L/h	52.09 (15.01)	97.24 (21.12)
V_z_/F, mean (SD), L	2,881.49 (1,042.38)	4,866.60 (1,504.81)

T_max_, time to peak plasma concentration; C_max_, peak plasma concentration; AUC_0-24_, area under the plasma concentration-time curve from time zero to 24 h; AUC_0-t_, area under the plasma concentration-time curve from time zero to time t; AUC_0-∞_, area under the plasma concentration-time curve from time zero to infinity; T_1/2_, terminal elimination half-life; CL/F, apparent clearance; V_z_/F, apparent volume of distribution; SD, standard deviation.

**TABLE 6:** Geometric mean ratios of pharmacokinetic (PK) parameters in the fasted and fed states.

PK parameter	Geometric mean		Geometric mean ratio	
	Fasted (*n* = 16)	Fed (*n* = 16)	Ratio	90%Cl
AUC_0-t_, h*ng/mL	376.99	193.72	0.51	0.48–0.56
AUC_0-∞_, h*ng/mL	399.23	211.18	0.53	0.49–0.57
C_max_, ng/ml	38.79	6.09	0.16	0.13–0.19

C_max_, peak plasma concentration; AUC_0-t_, area under the plasma concentration-time curve from time zero to time t; AUC_0-∞_, area under the plasma concentration-time curve from time zero to infinity.

Double peaks were observed in plasma concentration-time profiles of all single and multiple dose cohorts. The two peaks appeared at about 0.5–2 h and 4–6 h after dosing in all these cohorts with similar values presented.

In the metabolic transformation study, TQ-F3083 was detected in urine and feces samples. The 0–72 h accumulated excretion recovery percentages were 7.84% in urine and 5.76% in feces.

### PD Properties

**FIGURE 3:**
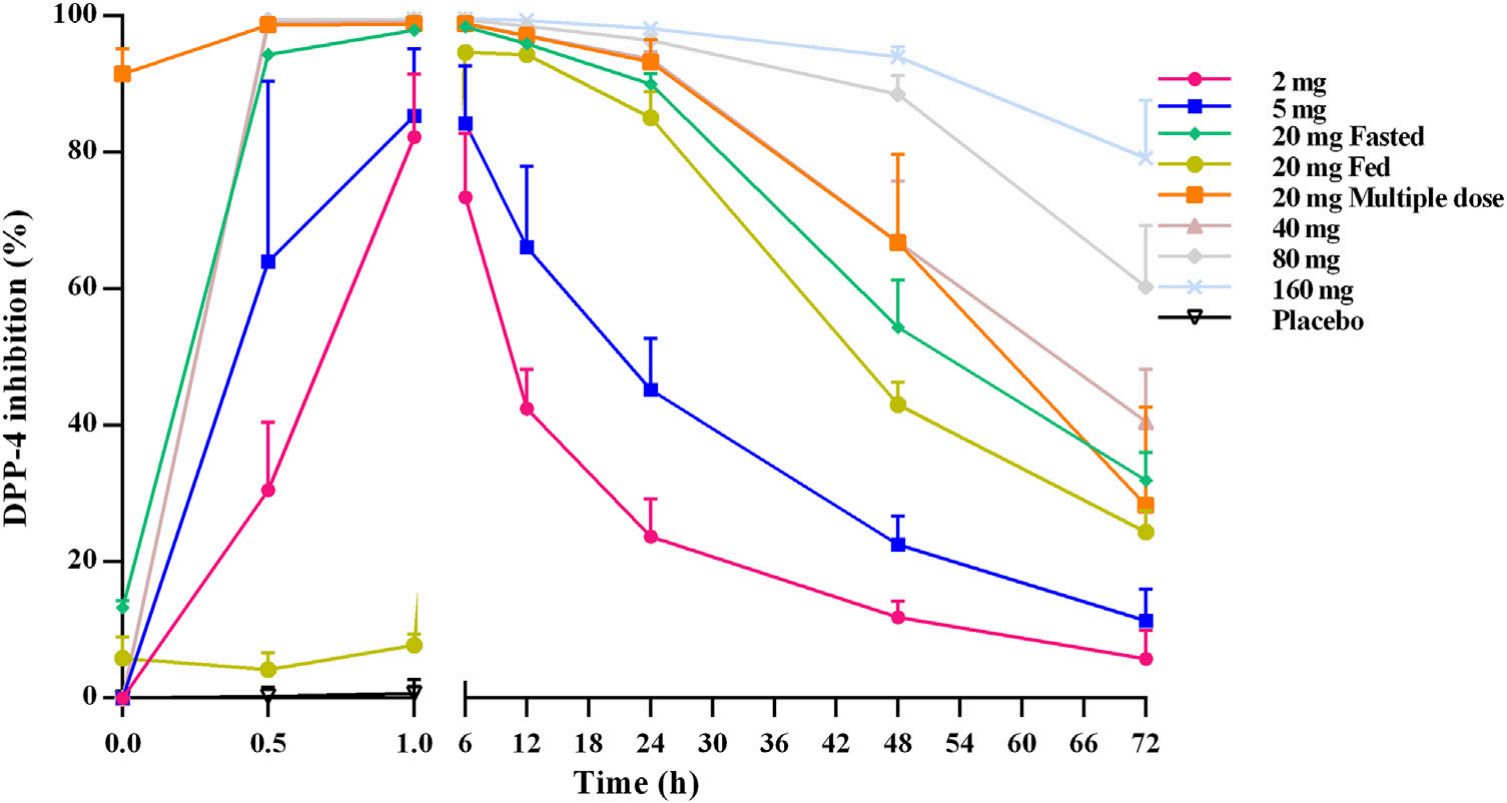
Mean (SD) DPP-4 inhibition–time profiles after oral administration of TQ-F3083 at doses of 2–160 mg.

Figure 3 shows the mean DPP-4 inhibition rate–time profile. DPP-4 activity was inhibited in a dose-dependent manner over the studied TQ-F3083 dosage range with mean maximal inhibition rates of 82.30–99.61%. In all cohorts, maximal inhibition was reached by 1 h after dosing, except in cohort 3 in the fed state, for which maximal inhibition was not reached until 6 h. The inhibition activity in all cohorts persisted to 72 h after dosing with inhibition rates ranging from 5.82 to 79.25%, and the >80% DPP-4 inhibition for 24 h was observed for all dose levels >5 mg. Compared to that achieved with an oral dose in the fed state, higher inhibition activity was observed in the fasted state, and multiple doses showed a similar profile to the single dose.

**FIGURE 4:**
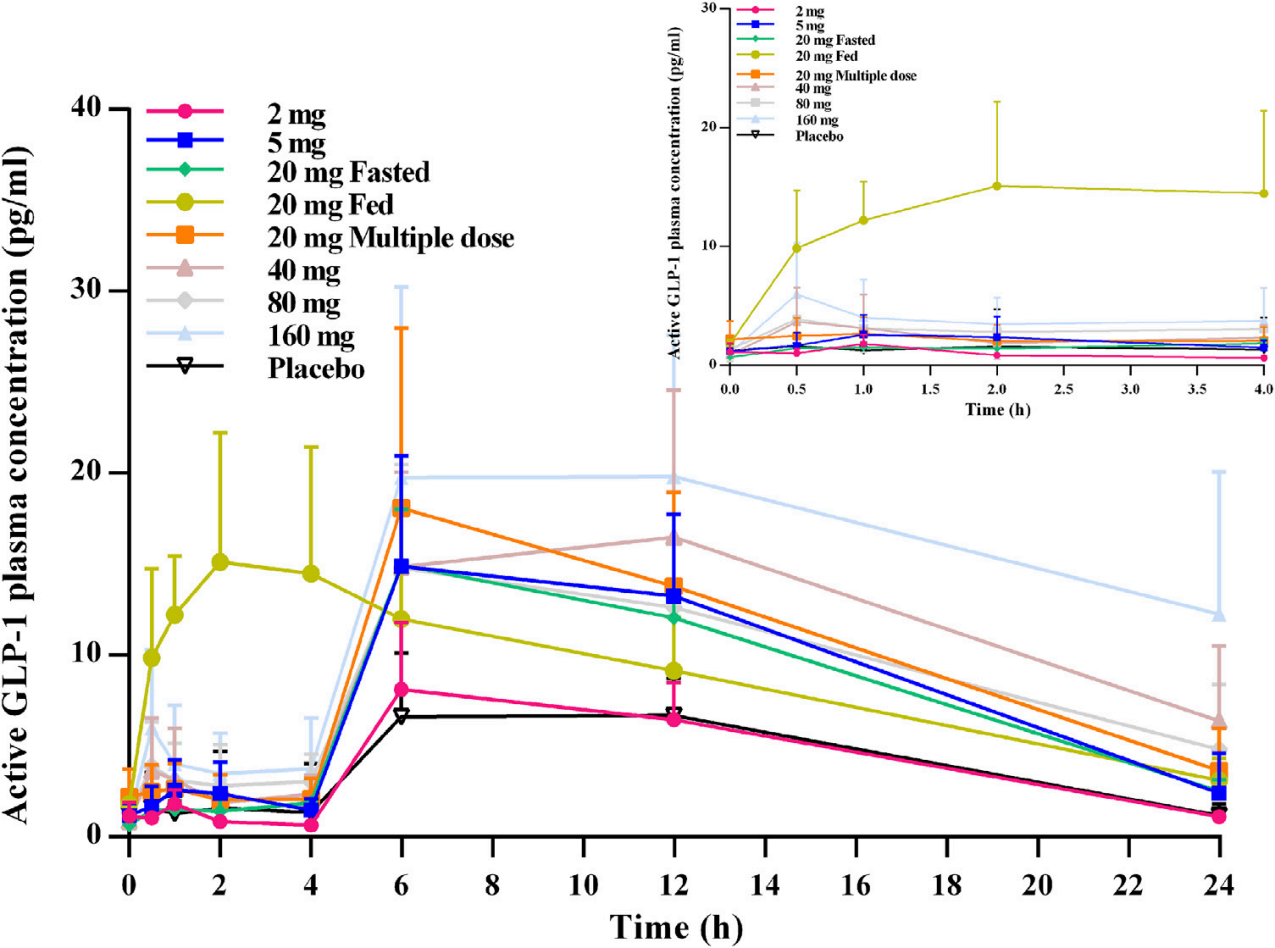
Mean (SD) active GLP-1–time profiles after oral administration of TQ-F3083 at doses of 2–160 mg.

From the active GLP-1–time profiles for all of the studied cohorts, an obvious bimodal curve was observed in the level of active GLP-1 (Figure 4). The peaks emerged 0.5–1 h and then ∼6 h after oral administration. In general, no obvious dose-dependent changes in the active GLP-1 concentration were observed.

The plasma concentrations of insulin, glucagon, and C-peptide also did not show significant relationships with oral administration of TQ-F3083.

### PK/PD Relationship

**FIGURE 5:**
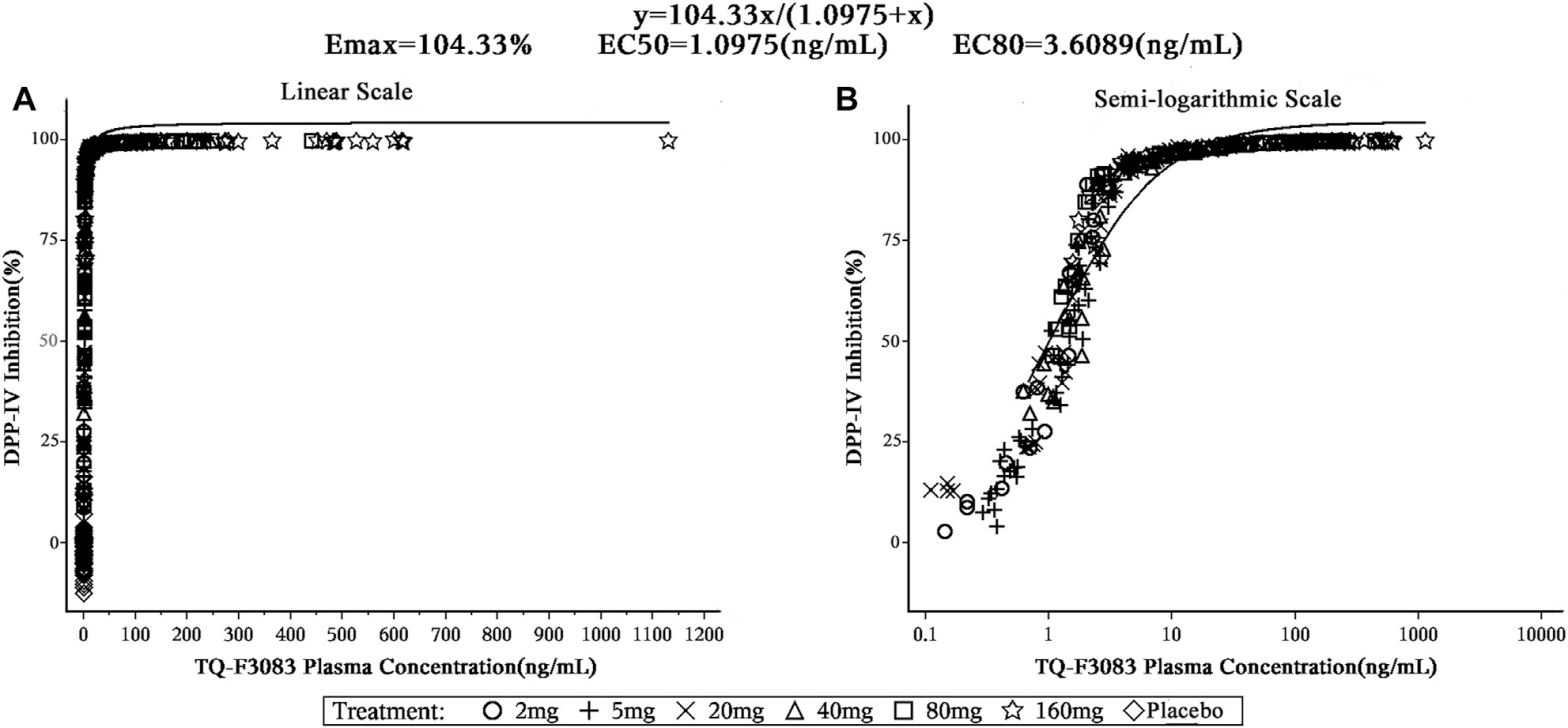
Correlation between plasma TQ-F3083 concentration and DPP-4 inhibition.

The TQ-F3083 concentration was satisfactorily fitted using the E_max_ model, and from this modeling, the 50% effective concentration (EC_50_) of TQ-F3083 in plasma was 1.10 ng/ml and the 80% effective concentration (EC_80_) was 3.61 ng/ml (Figure 5). The relationship between TQ-F3083 exposure (C_max_, AUC_0-t_, AUC_0-∞_) and the maximun DPP-4 activity inhibition rate was well fitted using the E_max_ model. As the exposure increased, the DPP-4 inhibition activity of TQ-F3083 increased and gradually approached the efficacy platform (Supplementary Figures S1–3).

## Discussion

The present phase I, randomized, double-blind, placebo-controlled clinical trial is the first to investigate the safety and PK and PD characteristics of single and multiple doses of TQ-F3083 capsules, as well as the corresponding food-effect. The demographic and baseline characteristics were similar across all the cohorts, and favorable tolerability results were observed after administration of a single dose of TQ-F3083 at concentrations ranging from 2 to 160 mg and multiple 20 mg doses with no MTD identified. Two grade 3 AEs of elevated triglyceride level were reported, with one each in the investigational product and placebo groups, and in total, only 2 of 50 subjects who were given TQ-F3083 suffered elevated triglyceride levels, suggesting that this is likely not a noteworthy AE for TQ-F3083. Other treatment-related AEs were mild or moderate, and no significant differences were observed between the treatment and placebo groups. No obvious dose-dependent AEs were reported, and no increase in the frequencies of AEs were observed in the multiple dose cohort, which also supports the safety of TQ-F3083.

Double peaks were observed in the plasma concentration–time profiles for both single and multiple doses. The two peaks appeared at about 0.5–2 h and 4–6 h after dosing. Also, in the SAD study, no dose-proportionate increase in C_max_ was observed. These results support that TQ-F3083 has a phenomenon of enterohepatic circulation. Having lunch stimulated the secretion of bile, the drug was excreted into intestinal lumen via bile and reabsorbed into plasma. The t_1/2_ of TQ-F3083 was verified to range from 25.52 to 41.62 h, which is shorter than that of linagliptin (t_1/2_ > 100 h), and no accumulation occurred during the multiple dose study, which supports the once daily oral administration of TQ-F3083. Obvious biphasic elimination was observed in the plasma concentration–time profiles for all of the studied cohorts, similar to the results for linagliptin (Retlich et al., 2010; Graefe-Mody et al., 2012), including a rapid decline followed by a mild elimination, suggesting the rapid distribution of TQ-F3083 from plasma to tissue and strong inhibition of DPP-4 by TQ-F3083 in tissue. According to the preclinical PD results in obese mice model of T2DM, the translated minimum effective equivalent dose was 17.5 mg (0.25 mg/kg, calculated based on the 70 kg body weight) in human. Considering the specifications of the capsules, 20 mg was chosen in the food effect and multiple dose studies to be close to the clinical effective dose. The food effect study showed that the PK exposure was decreased after a fed breakfast. It may because that food slowed down the rate of gastric emptying and increased the drug residence time in the stomach, which damaged the structure of TQ-F3083. And also the absorption of TQ-F3083 may be reduced as results of the changes of pH value in stomach and intestine after a fed breakfast. It was also demonstrated that oral TQ-F3083 in the fasted state was a more suitable method of administration for subsequent clinical studies in T2DM patients. In multiple dose study, the steady-state was reached after 4 days of continuous administration and no obvious accumulation was observed, which supported the safety of once daily oral administration. The 0–72 h accumulated excretion ratio of the parent drug was low, suggesting that TQ-F3083 is mainly eliminated in the form of metabolites. C-14 isotope tracer method will be employed to further explore the excretion balance of TQ-F3083. And the similar excretion percentages in urine and feces support that a renal impairment study to be conducted to verify the potential to administer TQ-F3083 in patients with renal function impairment.

DPP-4 inhibition by over 80% is considered to be clinically effective for DM patients (Herman et al., 2005; Sarashina et al., 2010). In the present study, TQ-F3083 provided >80% inhibition for 24 h at the 20–160 mg dose levels, supporting once daily oral administration for subsequent clinical trials. After oral administration of TQ-F3083 capsules, the DPP-4 inhibition activity increased from 0 to 1 h and persistent high DPP-4 inhibition activity was observed from 1 to 6 h in all the cohorts, then the activity gradually decreased from 6 to 72 h. The PD characteristic of TQ-F3083 was consistent with PK, demonstrating TQ-F3083 not an irreversible blockade of DPP-4 enzyme. The EC_50_ for TQ-F3083 was estimated to be 1.10 ng/ml (2.35 nM), which is slightly higher than that for linagliptin (1 nM) and much lower than those for other DPP-4 inhibitors on the market, such as sitagliptin (19 nM) and vildagliptin (62 nM) (Thomas et al., 2008; Gou, et al., 2019), demonstrating the good inhibitory activity of TQ-F3083. The GLP-1 concentration increased rapidly after meals, and therefore, it is more informative to explore the influence of TQ-F3083 on the active GLP-1 level in the first 0–4 h after dosing. In all of the studied cohorts except those given placebo, an increased in the active GLP-1 concentration was observed after TQ-F3083 administration, and greater than baseline active GLP-1 concentrations were observed at 4 h in all cohorts that received a dose of TQ-F3083 greater than 5 mg. Further clinical trials are warranted to explore the PD indicators in T2DM patients.

In conclusion, the current trial revealed that oral administration of a single dose of 2–160 mg TQ-F3083 and multiple 20-mg doses given once daily for 7 days were well tolerated. The PK and PD results support the use of oral administration of TQ-F3083 once daily in a fasted state for subsequent studies.

## Data Availability

The structure of the compound will be made available to scientists for the purposes of experimental verification. All other original contributions presented in the study are included in the article/Supplementary Material, further inquiries can be directed to the corresponding authors.
