# Supplementary figures and images for: First-in-Human, Double-Blind, Randomized, Placebo-Controlled Trial of TQ-F3083, a New Dipeptidyl Peptidase-4 Inhibitor, in Healthy Chinese Adults
Source: Front Pharmacol. 2021 Jul 22;12:689523. doi: 10.3389/fphar.2021.689523 (PMC8339258; doi:10.3389/fphar.2021.689523)

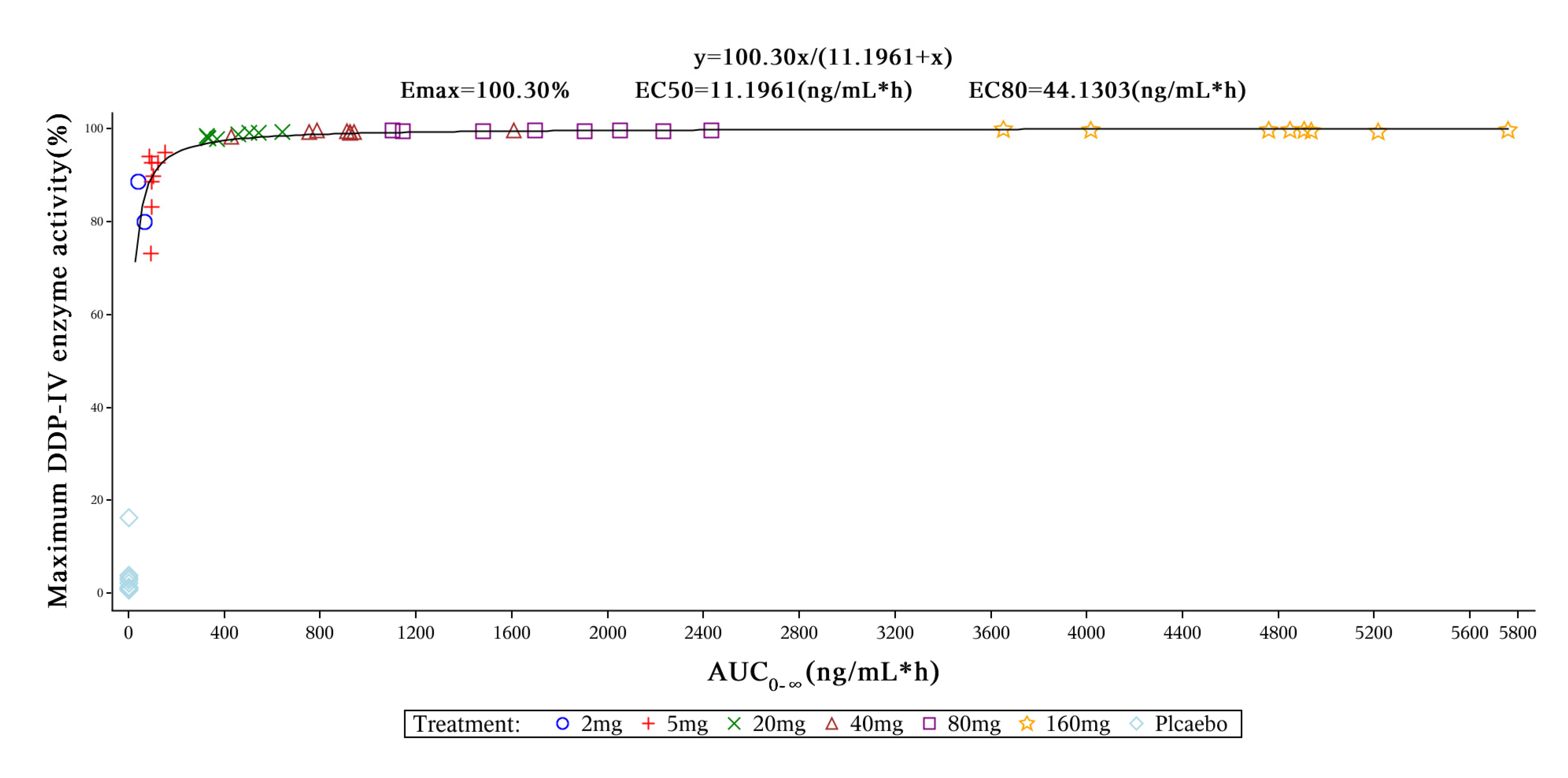

Supplement: Supplementary file 1 [file Image3.TIF]

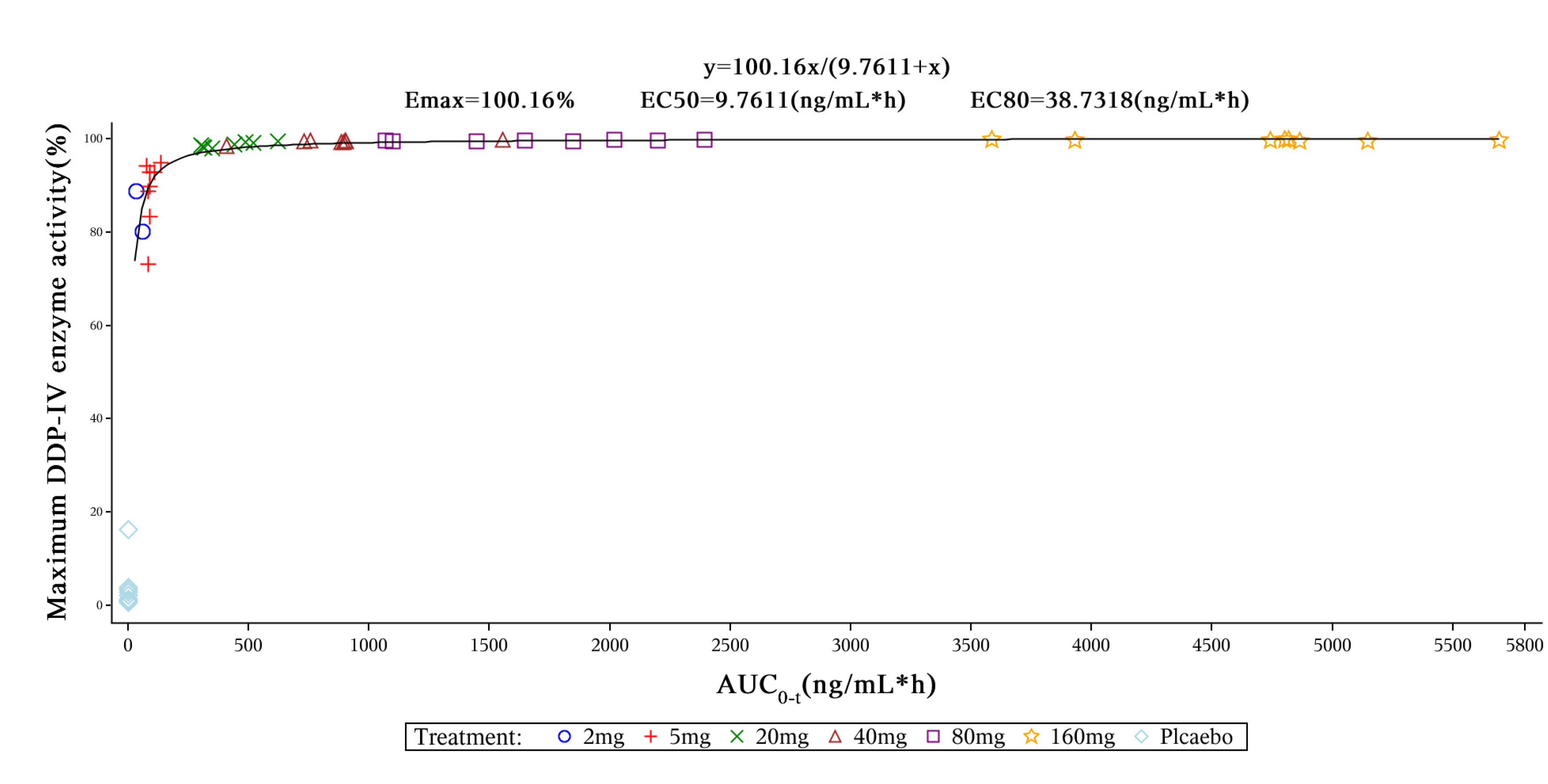

Supplement: Supplementary file 2 [file Image2.TIF]

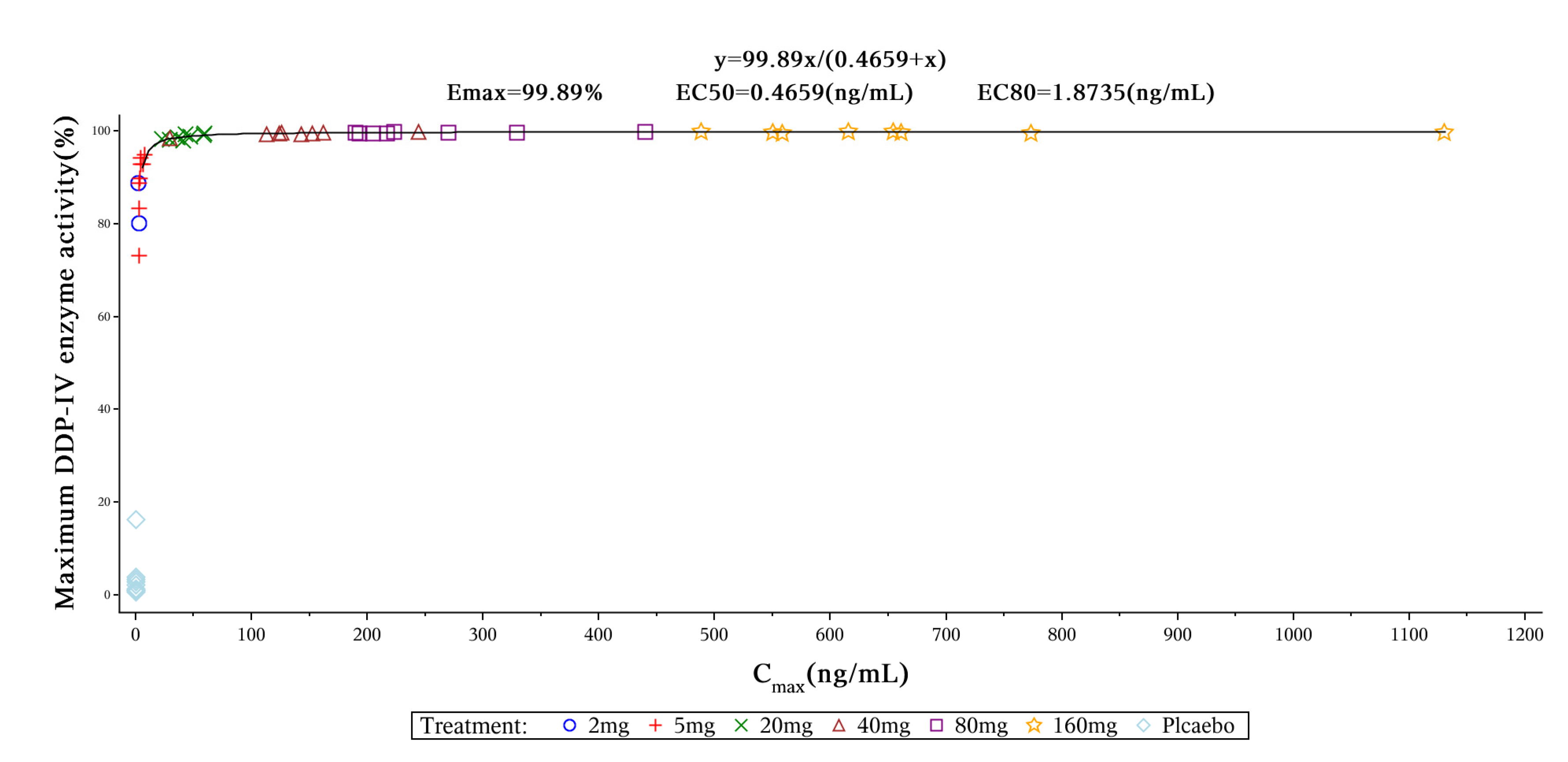

Supplement: Supplementary file 3 [file Image1.TIF]
